# Mapping Epidermal Growth Factor Receptor‑1 Sorting Domains in Endosomes with a Calibrated Three-Dimensional Expansion Microscopy Toolkit

**DOI:** 10.1021/acsnano.6c00277

**Published:** 2026-03-17

**Authors:** Tayla Shakespeare, Rajpinder S. Seehra, Neftali Flores Rodriguez, Nkolika Atuanya, Thomas M. D. Sheard, Ralf Köhler, Daniel Bose, Lydia Wunderley, Philip Woodman, Barbara Ciani, Izzy Jayasinghe

**Affiliations:** † School of Biosciences, Faculty of Science, 7315 The University of Sheffield, Sheffield S10 2TN South Yorkshire, U.K.; ‡ Division of Molecular and Cellular Function, Faculty of Biology, Medicine, and Health, Manchester Academic Health Science Centre, 5292 University of Manchester, Manchester M13 9PL, U.K.; § ITQB Nova, University of Lisbon, Av. da República, Oeiras PT 2780-157 Lisbon, Portugal; ∥ Sydney Microscopy & Microanalysis, 4334 University of Sydney, Sydney 2006 NSW, Australia; ⊥ School of Mathematical and Physical Sciences, Faculty of Science, The University of Sheffield, Sheffield S10 2TN South Yorkshire, U.K.; # EMBL Node in Single Molecule Science, Department of Molecular Medicine, School of Biomedical Sciences, Faculty of Medicine & Health, 7800 University of New South Wales, Sydney 2052 New South Wales, Australia

**Keywords:** endosomes, EGFR1, Rab5a, endofin, expansion microscopy, super-resolution, 3D visualization

## Abstract

Endosomes are nanoscale intracellular compartments that sort and recycle cell-surface receptors such as epidermal growth factor receptor-1 (EGFR1). Nanometer-scale interactions and coclustering of signaling proteins, cargo, and the membrane are critical to this process, yet direct 3D visualization has been hindered by the limited resolution of conventional and super-resolution microscopies. Here, we adapt expansion microscopy (ExM) to visualize and quantify nanoclusters of endosomal proteins in human retinal pigment epithelial (RPE-1) cells. We developed a 3D distortion analysis leveraging the Farneback optical-flow principle to detect anisotropies in hydrogel expansion, revealing under-expansion of cytoplasmic regions within ExM hydrogels and overestimation of size and distance measurements of small compartments such as endosomes. To calibrate ExM images of cytoplasmic regions containing endosomes, we introduced a self-assembling protein nanocage that reports the true local nanoscale expansion factor. To stimulate and visualize EGFR1 internalization and sorting, we applied a pulse-chase protocol with fluorescently tagged epidermal growth factor (EGF), fixed cells at 15 and 30 min, and subjected samples to 10-fold ExM and multiplexed 3D Airyscan microscopy to map cargo and EGFR1 relative to other endosomal proteins. A volume tracing pipeline was developed to visualize the changes in the labeled EGF and EGFR1 densities at the limiting membrane of the endosomes. These changes included enrichment of EGF and EGFR1 in the endosomal interior and accumulation of Rab5a near the limiting membrane during early endosome maturation. Together, this multiplexed 3D ExM toolkit provides a quantitative framework for visualizing and measuring small subcellular organelles at true molecular-scale resolution.

## Graphical abstract

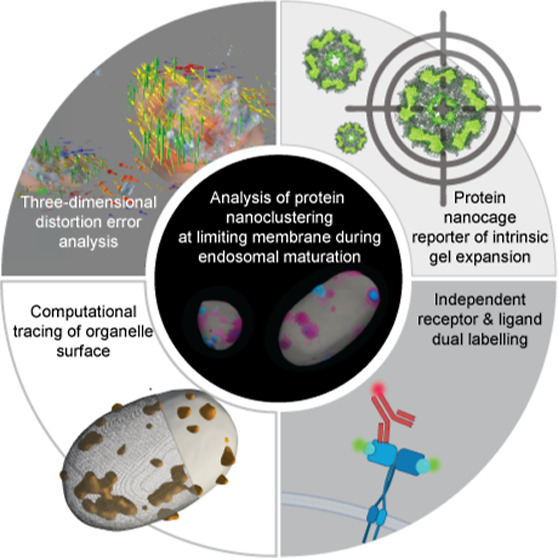

## Background

The spatial and temporal organization of membrane proteins within the endosomal system is critical for regulating cellular signaling, receptor trafficking, and membrane homeostasis. A prime example is epidermal growth factor receptor 1 (EGFR1), which, upon ligand binding at the plasma membrane, is rapidly internalized and trafficked through the endolysosomal system.^1^ They are sorted via small endosomal compartments and recycled back to the plasmalemma or trafficked to the lysosomes for degradation. This precise sorting process represents a critical decision point where cells must balance between terminating persistent signals and maintaining cellular responsiveness by regulating the surface receptor pool. Endocytosed EGFR1s often assemble into discrete nanoscale domains or nanoclusters.^2,3^ These domains are enriched for activated receptors and can recruit specific sets of effector proteins that modulate signaling duration, strength, and pathway specificity.

Multiple signaling and effector proteins orchestrate the dynamics and fate of EGFR1 nanoclusters. Rab GTPases, such as Rab5A and Rab7A, define the temporal identity of early and late endosomes.^4^ Rab5 activates the phosphoinositol 3-phosphate (PI3P) kinase Vps34 to generate local pools of PI3P^5^ and mediates the recruitment of downstream factors including early endosome antigen 1 (EEA1) via direct interaction and through the binding of the EEA1 FYVE domain to PI3P.^6^ PI3P helps recruit the ESCRT-0 component hepatocyte growth factor-regulated tyrosine kinase substrate (Hrs) to nanodomains that drive EGFR1 sorting to intraluminal vesicles (ILVs).^7,8^ The FYVE-domain protein, endofin (also known as ZFYVE16), further enriches these nanodomains, coupling phosphoinositide recognition to SMAD signaling and ILV sorting,^9,10^ The interactions of endosomal proteins are spatially regulated, yet their spatial organization and the nanoscale architectures of endosomes facilitating this process remain challenging to visualize with conventional microscopies.

Endosomes are typically 100–300 nm in diameter across their limiting membranes; many of the finer membrane topologies such as tubules and intraluminal vesicles are only tens of nanometers in scale.^11,12^ Diffraction-limited optical microscopy (with lateral and axial resolution limits of ∼250 nm and ∼600 nm, respectively) cannot resolve individual endosomes, nor can it distinguish protein nanoclusters that may be separated by <100 nm. Even conventional super-resolution techniques such as STORM, PALM, or SIM, while offering improved spatial resolution, remain inherently unsuitable for 3D volumetric imaging of densely packed cellular compartments. 3D implementations of STORM or PALM, such as biplane or astigmatism-based methods, could improve the axial resolution to ∼50–70 nm, allowing the visualization of protein nanoclusters around small organelles such as clathrin-coated pits.^13^ Alternatively, combining 2D protein localization with tomographic electron microscopy (superCLEM) provides a ground truth for molecular localization of protein clusters and cargo into subendosomal domains such as recycling tubules that may return the proteins to the plasmalemma.^11^ While sub-10 nm localization techniques such as DNA-PAINT have emerged as powerful tools for multiplexed analysis of endosomal markers,^14^ they remain best suited for near-field imaging of flat and/or relatively sparsely labeled samples. These types of image data therefore remain principally limited to 2D.

Expansion microscopy (ExM) overcomes many of these limitations by physically inflating the sample prior to imaging, decoupling resolution from the limits posed by diffraction.^15^ When combined with confocal microscopy, ExM enables isotropic resolution below ∼70 nm,^16^ even in optically thick samples such as tissue sections and whole organisms.^17–19^ With structured illumination or Airy-scanning imaging, effective resolutions of 15–30 nm can be routinely achieved.^16,20^ Critically, ExM enhances both lateral and axial resolution, making it ideally suited for resolving nanoclusters within volumetric compartments, such as endosomes. Variations of ExM chemistry over the past decade have also allowed different degrees of expansion, differential targeting of biomolecule classes (e.g., proteins, lipids, or nucleic acids),^21–23^ and/or different degrees of expansion isotropy.^24,25^

A key barrier to widespread adoption of ExM for quantitative molecular imaging lies in its spatial fidelity. Physical expansion can be heterogeneous, leading to distortions and variable expansion factors (EFs) across regions of interest. Errors in ExM can manifest either as distortions of the sample ultrastructure or under-expansion^26^ that can limit the quantitative nature of ExM image data. Emerging solutions for this include registration of post-ExM images against a pre-ExM image,^27,28^ imprinting the gels with fiduciary patterns,^29^ and statistical analysis of intrinsic ultrastructures such as microtubules and sarcomeres.^20,30^ However, the lack of a truly nanoscale reporter of the expansion of protein-rich cellular ultrastructure has remained a key barrier to its adoption for visualizing subcellular compartments.

In this paper, we present a quantitative framework for the 3D ExM imaging of small intracellular compartments with a focus on the nanoscale organization of EGFR1 sorting within the endosomal system. To verify the degree of expansion of subcellular structures, we have developed a 3D error detection analysis pipeline and adapted a genetically encoded self-assembling protein nanocage as an intrinsic calibrant to the local EF. By leveraging this quantitative 3D ExM imaging approach, we have mapped the distribution of activated EGFR1 and epidermal growth factor (EGF) ligands within the maturing endosome relative to key sorting effectors, including Rab5A and endofin, at specific time points following endocytosis.

## Results and Discussion

### 3D Expansion Microscopy for Imaging Endosomal Proteins

We adopted ExM for visualizing the 3D organization of endosomal proteins within human retinal pigment epithelial (RPE-1) cells. Proteins associating with the endosomal membrane, immunolabeled in situ, were subjected to the expansion protocol, which consisted of the principal steps of anchoring and gelation, digestion, and the subsequent expansion by hydration with deionized water (dH_2_O; schematically summarized in Figure 1A). In Airyscan images of unexpanded cells, endosomes labeled for early endosomal antigen-1 (EEA1) commonly appeared as bright, diffraction-limited puncta in perinuclear regions (Figure 1B). By comparison, similar cell samples expanded with 10-fold (10×) ExM and imaged with the resolution-enhancing Airyscan^31^ featured noticeably more torus-shaped labeling patterns that reflected the endosomal surface localization of EEA1 (Figure 1C). On close examination of images, we confirmed that the smaller endosomes (⌀ < 200 nm) were unresolved, while a ring-like morphology was present only in larger (⌀ > 300 nm) endosomes in Airyscan images of unexpanded samples (magnified views shown in Figure 1D-i,E-i). In samples expanded with 4× ExM and imaged with Airyscan, the luminal spaces of both small and large endosomes were discernible. However, the boundary of EEA1 labeling on the endosomal surface appeared dense and continuous (Figure 1D-ii,E-ii). By comparison, in samples expanded 10-fold, the EEA1 labeling followed a more discernibly punctate morphology on the endosomal surface (Figure 1D-iii,E-iii). This punctate morphology resembled either individual markers or small clusters of well-resolved individual antibody markers observed before in other cell types by combining 10× ExM with Airyscan.^20^

**Figure 1:**
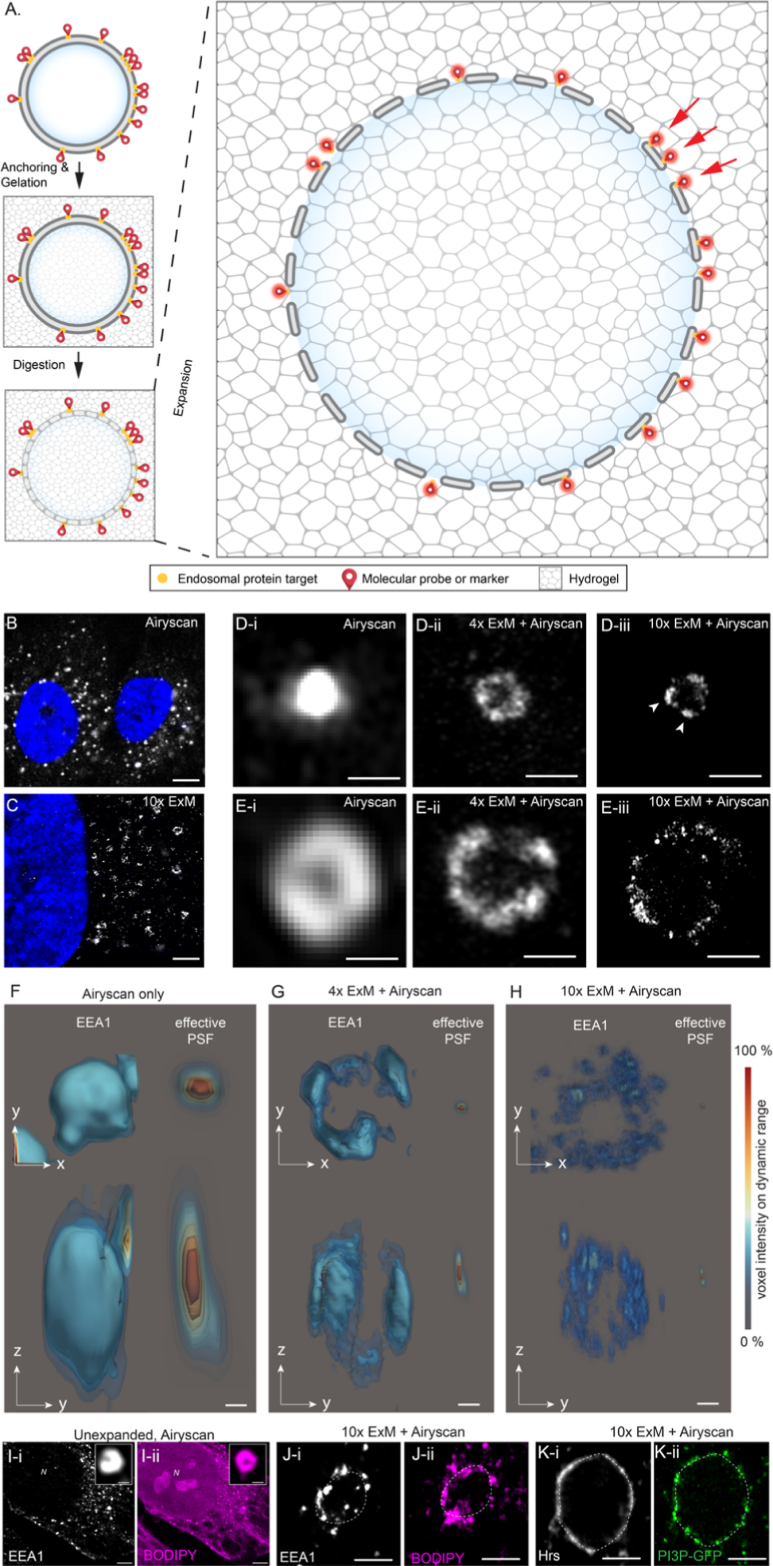
3D expansion microscopy of endosomes. (A) Schematic diagram of ExM of endosomal proteins. In this method, protein targets in the cells are labeled and chemically anchored before the hydrogel is polymerized in situ. Following a mild enzymatic digestion, the molecular-scale structure and markers imprinted onto the hydrogel matrix are expanded by osmotic swelling to physically inflate the samples. Previously unresolvable structures are now resolved due to the physical separation of their markers (red arrows). (B) Confocal image of unexpanded RPE-1 cells labeled for EEA1 (gray) and nuclear stain DAPI (blue). (C) Airyscan image of an identical sample following 10-fold expansion (10× ExM) reveals the EEA1 endosomes as ring-like structures compared to diffraction-limited puncta. Magnified confocal images of (D-i) small and (E-i) large endosomal vesicles (⌀ < 100 nm and >300 nm, respectively), labeled for EEA1, illustrate the poorly resolved nature of the spherical endosomal shapes. By comparison, 4× ExM Airyscan images show both (D-ii) small and (E-ii) large endosomes of equivalent sizes as torus-shaped morphologies. With the superior resolution achieved with 10× ExM combined with Airyscan nanoclusters (arrowheads), EEA1 labeling was observed on the surfaces of both (D-iii) small and (E-iii) large endosomes. 3D isosurface contour rendered examples of endosomes from (F) unexpanded, (G) 4× ExM, and (H) 10× ExM RPE-1 cells labeled for EEA1 are shown in both *x*–*y* (upper) and *z*–*y* (lower) views. Shown on the right in each panel are the *x*–*y* and *z*–*y* views of the effective PSF (relative to the scale-corrected endosomes) as a result of the expansion. The color scale reflects the % of the voxel intensity across the full dynamic range of the structure. (I) Airyscan images of unexpanded cells costained with (I-i) EEA1 antibody and (I-ii) BODIPY630 NHS ester showed individual endosomes stained in both channels (magnified view in insets). (J) 10× ExM image of a similar cell indicating high density of staining encircling endosomes. (J-i) EEA1 punctate densities at the endosomal limiting membrane (gray). (J-ii) BODIPY staining (magenta) matched the endosomal shape reported by EEA1. (K) Equivalent comparison of (K-i) membrane-associated protein Hrs (gray) and (K-ii) endosome-specific PI3P-GFP labeling (green) both feature punctate staining densities at the endosomal limiting membrane. Scale bars: (B): 5 μm, (C): 2 μm, (D,E): 200 nm, (F–H): 100 nm, (I) (main): 1 μm, (I) (inset): 250 nm, and (J–K): 250 nm.

The resolution improvement achieved from ExM is the result of effective downscaling of the point spread function (PSF). To estimate the PSF of the Airyscan protocol used in our experiments, we imaged polystyrene microspheres with ⌀ 100 nm, averaged between 10 copies. Examples of exemplar endosomes from RPE-1 cells are compared between unexpanded, 4× ExM, and 10× ExM (Figure 1F–H). To scale, the effective PSF is shown in each example. This comparison reveals that the nanoclustering morphology on the endosomes, on both the top and lateral sides, is only resolved as the PSF is scaled below the typical size of each nanocluster. This is principally achieved with 10× ExM, as the effective size (reflected by the full-width at half-maximum); of the PSF approaches ∼17.5 nm laterally and ∼65 nm axially. Features of the Airyscan volumes of 10× ExM samples were the hollow interior of endosomes and the intricate patterns of EEA1 nanoclustering visible on the bottom, lateral, and top surfaces (Figure S1). While the intensity and density of the lateral nanoclusters were higher due to the poorer axial resolution and the greater axial signal integration, the pattern of nanoclusters on the top surface was best resolved in glancing, in-plane optical sections of the bottom surfaces of these endosomes (Figures S1 and S2).

In addition to antigen markers of endosomes, counterstains are highly desirable in visualizing these compartments. In double staining experiments, we observed that the NHS version of BODIPY630, shown previously to be attracted to lipid-rich membrane compartments,^32^ stains endosomes in high intensity (and verified by costain of EEA1; Figure 1I). However, as a nonspecific lipophilic stain, it also highlights other membrane-bound compartments. With ×10 ExM, we observe that BODIPY630 reports the same endosomal shape as EEA1 (Figure 1J). We also demonstrate phosphatidylinositol 3-phosphate lipid (PI3P)-targeted GFP staining as an alternative counterstain, also verified as endosome-specific by coimmunostaining for an endosomal membrane-associated protein recruited to the limiting membrane, Hrs. PI3P labeling is discontinuous, consistent with PI3P localizing to subdomains of the endosome limiting membrane that are also occupied by FYVE domain proteins that combine with Rab5a (Figure 1K).

### Tools for Spatial Error Analysis in ExM of Endosomes

A key limitation of ExM is that anisotropic gel expansion can lead to distortions of the ultrastructure and/or incorrect estimation of the EF in the region of interest. To observe distortions, ExM hydrogels of RPE-1 samples immunolabeled for EEA1 within bespoke, geometry-preserving microplates^26^ were imaged both prior to and after expansion. The square geometry of the hydrogels polymerized and expanded within each well of the microplate allowed us to record *x*–*y* coordinates of cells and structures of interest prior to expansion and then to efficiently track and reimage the same structure postexpansion (see details in Supplementary Methods Section 3.1). Figure 2A illustrates scaled and aligned 2D overlays of pre- and postexpansion Airyscan images (red and cyan, respectively) of a cell subjected to a 4× ExM experiment. The overlaid field of arrowheads reports the local distortion shift fields in the *x*–*y* planes. While most endosomes aligned with high fidelity (e.g., Figure 2A-i), some endosomes in the same image appeared more significantly shifted (Figure 2A-ii; arrows indicate shift vectors). The aligned images were subsampled, and the root-mean-square of the error (RMSE) was calculated from the resultant distortion shift vectors for each sampling scale. Figure 2B plots the RMSE across different length scales (where distance values reflect the post-ExM scale) ranging from 50 nm to 2.5 μm (Figure 2B), indicating a distortion error of ∼1–5% in the typical measurement range for 200–1000 nm.

**Figure 2:**
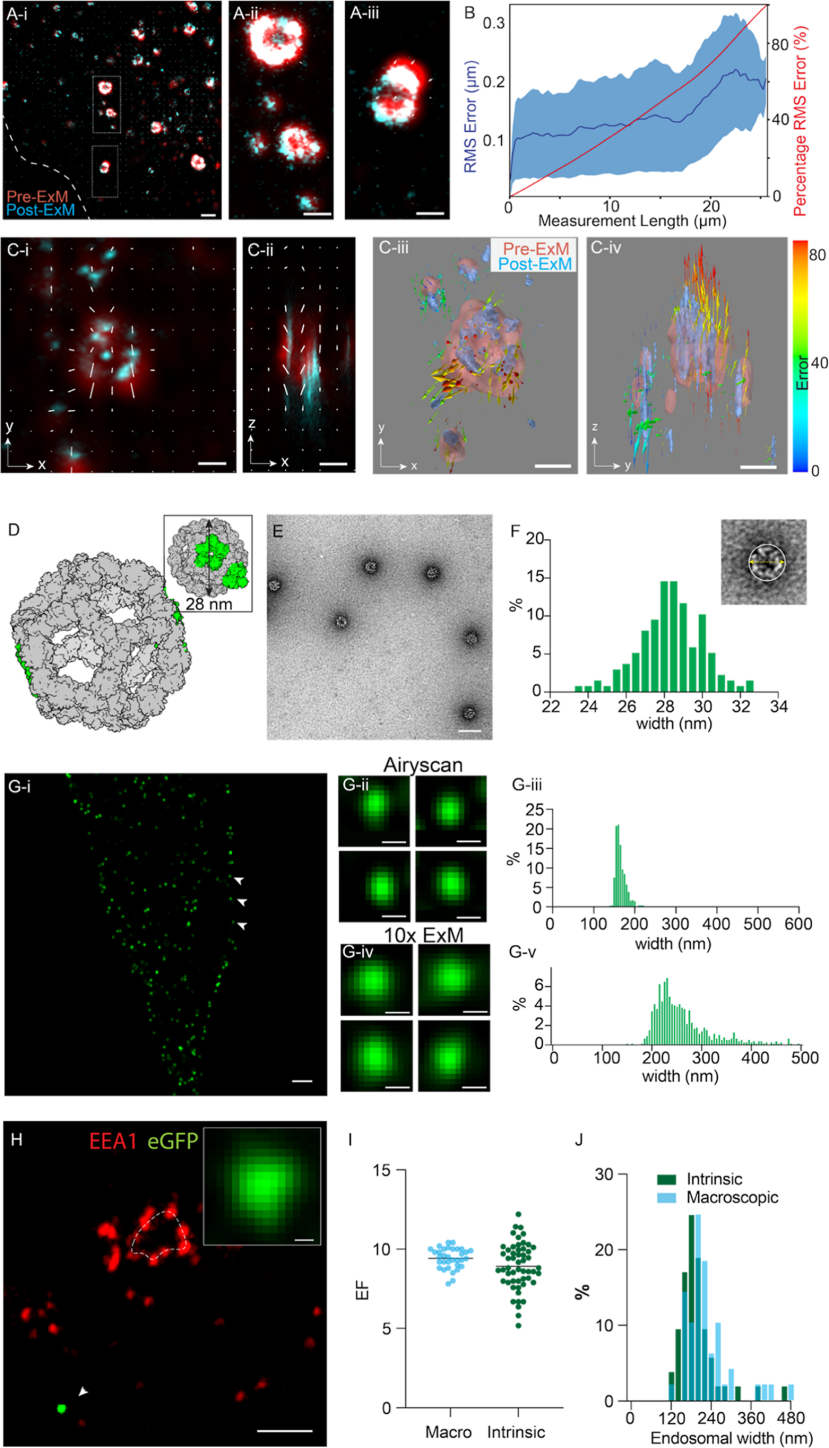
Calibrated expansion microscopy of endosomes. (A-i) Image of EEA1 labeling in a RPE-1 cell shows the pre- (red) and post-ExM (cyan) images overlaid with the distortion vector field (white arrows indicating the magnitude and direction of shift). Magnified views of endosomes indicate most endosomes show negligible registration errors (A-ii), while a minority of regions indicate shifts of ∼1% in magnitude (A-iii). (B) The plots of RMS error (mean curve across 6 data sets shown in dark blue; SD in light blue shading) and cumulative percentage error at different length scales of the image derived from this distortion analysis. (C-i) and (C-ii) are *x*–*y* and *x*–*z* views of a volumetric alignment of an endosome imaged before (red) and after (cyan) 10× ExM. The white arrows indicate the distortion vector components in each view. (C-iii) and (C-iv) illustrate isosurface rendering of the same volume from the same respective points of view. The color scale (right) indicates the magnitude of each distortion vector. (D) To report the intrinsic expansion factor, a self-assembling decahedral 60-subunit nanocage (gray) with GFP on each subunit (green) with an overall estimated diameter of ∼28 nm was expressed in the cells. (E) Negative-stain electron micrograph of purified nanocages containing two sfGFP per subunit, indicating their self-assembly. (F) A percentage histogram of the diameter of nanocages estimated with negative stain EM (illustrated as per the inset). (G-i) Fluorescence Airyscan micrograph of the RPE-1 cell (boundary indicated by the dashed line; DAPI-stained nucleus in blue) expressing individual nanocages containing one eGFP per subunit (green; arrowhead). Compared are magnified views of exemplar nanocages imaged with Airyscan microscopy, pre-ExM (G-ii) and post-ExM (G-iv). Histograms illustrate width analysis of the nanocages in unexpanded Airyscan images (G-iii) and post-ExM Airyscan images (G-v). (H) A 10× ExM image of a RPE-1 cell stained for EEA1 (red) observed adjacent to an intrinsically expressed nanocage (green; magnified view in the inset). (I) Dot plot comparing estimates of the expansion factor (EF) by macroscopic measurements (mean ± SD: 9.4 ± 0.6; *n* = 33 replicates) and intrinsic measurements based on mean nanocage widths (mean ± SD: 8.9 ± 1.4; *n* = 53 replicates). (J) Overlaid histograms of estimated endosomal width from data calibrated for macroscopic EF (cyan) and intrinsic EF (green). Scale bars: (A-i): 200 nm, (A-ii,A-iii): 100 nm, (C,D): 100 nm, (E): 50 nm, (G): 200 nm, (H): 200 nm, (K)-inset: 10 nm.

To further investigate the 3D nature of the distortions, arising particularly in experiments with a higher EF, we developed a 3D distortion mapping approach. A block-matching approach for calculating a transformation matrix was used for rescaling and aligning the pre- and post-ExM image volumes of the same region of interest (see details in Supplementary Methods Section 3.2; Figures S11 and S12). Registered image volumes were resectioned into single 2D planes, both in *x*–*y* and *x*–*z*. An adaptation of Farneback’s optical flow technique allowed us to calculate the 2D distortion shift vectors for a given point in both *x*–*y* and *x*–*z* planes (see details in Supporting Information Section 3.3). Figure 2C illustrates in-plane (*x*–*y*, Figure 2C-i) and axial (*z*–*x*, Figure 2C-ii) views of the pre- and post-ExM volumes containing one endosome (red and cyan, respectively), overlaid with the local distortion shift vectors (white arrows). The orthogonal shift components were used for calculating the true vectors, shown as arrows in Figure 2C-iii,iv. Radially oriented shift vectors (also see Figure S3) were commonly observed at the endosome in spite of good registration of the overall cell. The localized alignment error reflected under-expansion of the small compartments like endosomes, post-ExM (shown with a blue isosurface in panels C-i to C-iv, compared to the pre-ExM features (red).

### Protein Nanoscale Reporter of Intrinsic Gel Expansion

The sensitivity of this approach to distortion detection is ultimately limited by the resolution of the pre-ExM image. A truly nanoscale reporter of cellular expansion is required to verify cytosolic under-expansion. We adapted a homomultimeric 60-subunit decahedral protein nanocage developed previously by David Baker’s laboratory^33^ (Figure 2D) as a calibrant of the local gel expansion. The cDNA for the I3-01_60eGFP monomer of the nanocage was cloned onto a mammalian vector before transfecting the RPE-1 cells, which then expressed each subunit of the nanocage, which consisted of eGFP, self-assembled into a 60-meric nanocage with a ⌀ ≈ 28 nm (inset), observable throughout the cytoplasm. Negative stain transmission electron microscopy (TEM) was used to measure the widths of individual nanocages isolated and purified from similar cells (Figure S5). These measurements were comparable to those of nanocages purified and filtered from *E. coli* expression systems (Figures 2E and S4). The histogram in Figure 2F illustrates the distribution of the widths of purified nanocages (mean ± SD: 28.3 ± 1.6 nm; *n* = 138 nanocages; examples of TEM images at different stages of purification are shown in Figure S5). In Airyscan images of unexpanded RPE-1 cells, the eGFP fluorescence of the nanocages was observed as bright and diffraction-limited spots throughout the cell (arrowheads; Figure 2G-i). Close examination of the spots in these Airyscan images (magnified view of examples shown in Figure 2G-ii) and the subsequent width measurements (histogram in G-iii with a mean ± SD of 165.6 ± 12.8 (*n* = 678 nanocages; 3 cells)) confirmed this. Analysis of the cytoplasmic nanocages in Airyscan images following 10× ExM revealed larger, supra-resolution spots (Figure 2G-iv,v) and a broader distribution of widths (mean ± SD: 263.7 ± 63.1 nm; *n* = 794 nanocages; 7 cells). The latter was consistent with local heterogeneity in the expansion of the nanocages throughout the cell volume. Figure S6 compares the observed expansion of nanocages between different expansion protocols: 4× ExM, 10× ExM, and 10-fold Robust expansion (TREx),^34^ with the latter two able to resolve the fully expanded nanocage. The identity of the GFP-linked to the nanocages was briefly confirmed by secondarily labeling with an *anti*-GFP Alexa568 nanobody (Figure S6-I). When the transfected cells were immunolabeled for EEA1, the nanocages were commonly observed in regions adjacent to endosomes and within the same image planes (Figure 2H). To calibrate the local average EF, we divided the mean width of the nanocages in post-ExM Airyscan images by the mean width of the nanocages estimated with TEM. A comparison of the intrinsic EF estimates based on nanocage measurements against the macroscopic EF estimates (by measuring the dimensions of the hydrogel before and after 10× ExM expansion) across 33 samples is shown in the dot plot in Figure 2I. While the intrinsic EF measurements displayed greater variability (SD of 1.4 compared to 0.6), they also showed ∼6% lower mean (*p* = 0.04; df = 86; one-tailed Mann–Whitney U test). Figure 2J illustrates the left-shift in a histogram of endosomal widths (a median drop of 20.3 nm; *p* < 0.05, df = 100; Wilcoxon test) measured from 10× ExM Airyscan data once the intrinsic EF was used to calibrate the image instead of the macroscopic EF (see similar analysis for 2D area of endosomes in Figure S7).

### Visualization of EGF and EGFR1 Sorting Using ExM

To visualize endosomal sorting of EGFR1, we performed a series of pulse-chase experiments employing Alexa Fluor^488^-conjugated streptavidin bound to biotinylated EGF ligand. The cells were fixed at 15 and 30 min time points following the EGF pulse, allowing the internalized EGF/EGFR1 complexes to be sorted via early endosomes and their maturation into multivesicular bodies (Figure 3A). Following fixation, EGFR1 was additionally immunolabeled, allowing us to simultaneously visualize the spatial distribution of the receptor and the ligand (Figure 3B). Unexpanded Airyscan images showed bright, diffraction-limited spots of EGFR1 labeling (magenta) throughout the cytoplasm of the cells. Closer examination of magnified images revealed colocalization of discrete EGF puncta with the EGFR1 outline of larger endosomes. ExM Airyscan images of endosomes from cells fixed at 15 min revealed discrete nanoscale domains of EGFR1 clustering along the boundary of the endosome (indicated with a dashed line; Figure 3E-i). Both EGFR1 and EGF were often localized to the vesicular interior in ExM Airyscan images of endosomes, particularly at 30 min (Figure 3F-i). 3D data sets of the 15 min time point were isosurface rendered and visualized in orthogonal views (Figure 3E-ii,iii) to reveal the overwhelming localization of EGF and EGFR1 to the endosomal surface. By comparison, ExM Airyscan volumes from 30 min clearly indicate a greater subset of EGF/EGFR1 nanoclusters localized in the center of the vesicle (example shown in orthogonal views in F-ii vs F-iii). To analyze the spatial relationships between the ligand and receptor labels, calibrated ExM Airyscan data sets were subjected to a segmentation using Huygens software, allowing us to discretize and localize individual puncta in both EGF and EGFR1 channels (illustrated schematically in Figure 3G). The violin plot comparing the measured widths of the segmented puncta (Figure 3H) led to two key observations from this analysis. First, the EGFR1 puncta were consistently wider than the EGF puncta at both time points (by 84% at 15 min and by 34% at 30 min). Second, the EGF puncta at the 30 min time point were ∼38% wider than that at 15 min (mean ± SD of 44 ± 25 nm vs 32 ± 22 nm), particularly observed in the endosomal interior. Analysis of the nearest neighbor distance of each EGF-to-EGFR1 puncta revealed an ∼60% increase in the ligand–receptor marker distances from 42 ± 26 nm to 67 ± 30 nm between 15 and 30 min time points (Figure 3I). In contrast, the ratios between the total volume measurements at the two time points (Figure 3J) and the ratio between the total number of puncta at the two time points (Figure 3K) were consistently lower and less variable at the 30 min point. This indicates a progressive enrichment of ligand-bound EGFR1 within the endosome as well as possible recycling out of the endosome.

**Figure 3:**
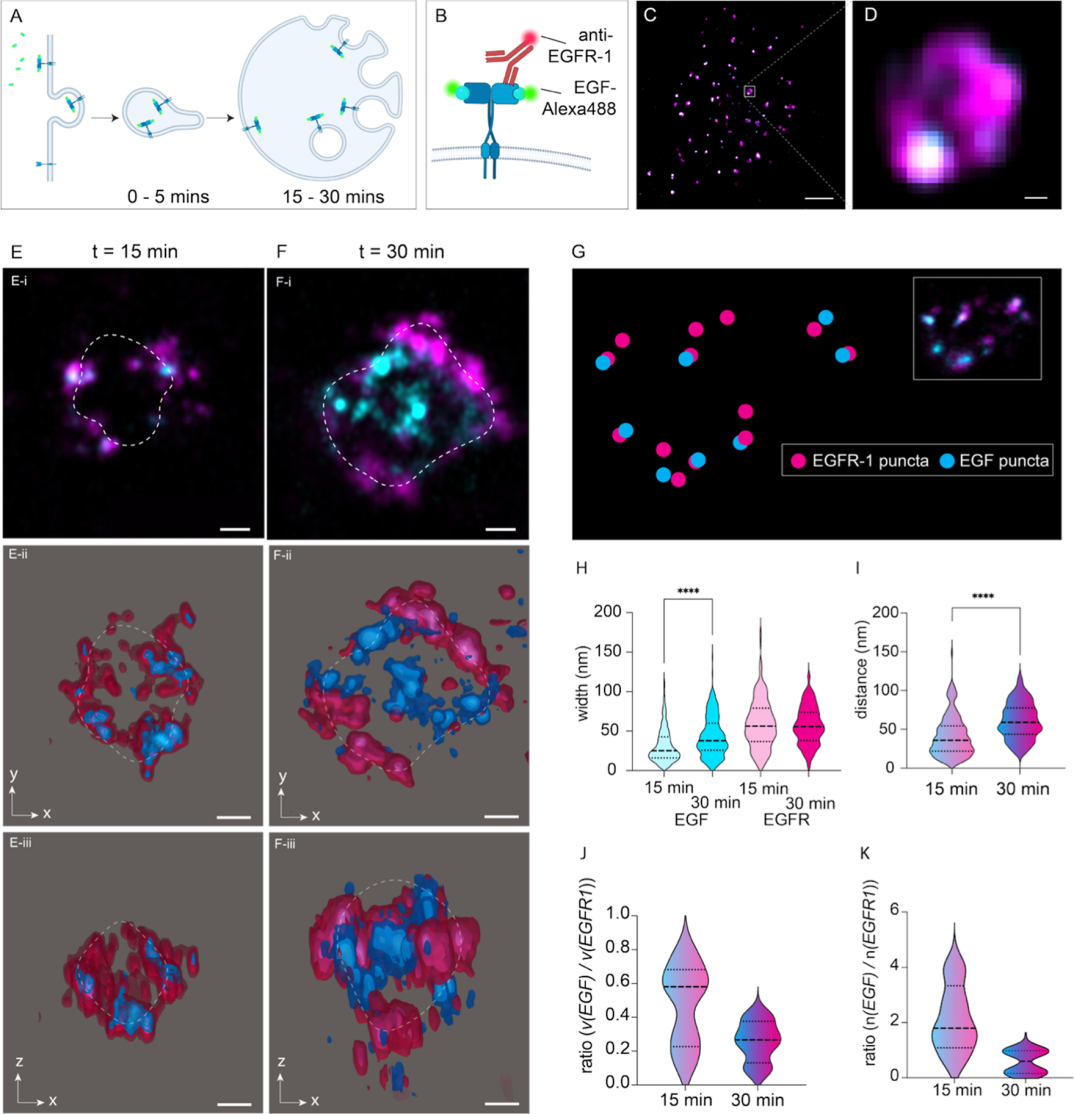
Nanoscale measurement of EGFR1 sorting during endosomal maturation. (A) Schematic illustration of EGF stimulation leading to endocytosis of the EGF ligand-EGFR1 receptor complex into early, small endosomes and the subsequent sorting of EGFR1 during the maturation and expansion of the endosome over the time scales of 15–30 min (t). (B) Illustration of the fluorescent pulse-chase dual imaging of the Alexa 488-EGF and posthoc immunolabeled EGFR1 receptor allowing independent localization of EGF and EGFR1. (C) Airyscan overview image of a cell with dual EGF-EGFR1 labeling (cyan and magenta, respectively). (D) Magnified view of an endosome in panel (C), exemplary of the toroid morphology of EGFR1 staining (magenta) and confined and colocalized puncta of EGF (cyan). 10× ExM images of EGF and EGFR1 at exemplar endosomes at pulse-chase time points of (E-i) 15 min and (F-i) 30 min, noting the additional densities of EGF and EGFR1 localization in the middle of the vesicle at the latter. The dashed white line indicates the approximated boundary of the endosomal labeling. Orthogonal views of isosurface visualization of EGF (blue) and EGFR1 (magenta) of the same endosomes from 15 min (E-ii,iii) and 30 min (F-ii,iii) time points. (G) To analyze the relationship between puncta of EGF and EGFR1, the punctate densities were segmented, and the nearest neighbor distance from each EGF punctum to the nearest EGFR1 punctum was analyzed (the corresponding original image is shown in the inset). (H) Violin plot comparing the width of the EGF (cyan) and EGFR1 (magenta) puncta at 15 min (light colored) and 30 min (dark colored) time points. Comparisons: **** from the Mann–Whitney test. *p* < 0.0001, df = 696 puncta. (I) Violin plot of the measured nearest neighbor distance from EGF puncta to nearest EGFR1 ****p < 0.0001, df = 213 puncta. Also shown are violin plots of (J) the ratio between the total volume of EGF puncta and the total volume of EGFR1 puncta and (K) the ratio between the number of EGF and EGFR1 puncta in each endosome at 15 min (*n* = 9 endosomes, 9 cells) and 30 min (*n* = 9 endosomes, 8 cells). Scale bars: 50 nm.

### Multiplexed, 3D ExM of Ligand, Receptor, and Sorting Proteins

Rab5a is recruited to the endosomal surface and organized into nanodomains by the Rab5 guanine nucleotide exchange factor (GEF) complex of Rabex-5 and Rabaptin-5.^35–37^ Similarly, endofin is recruited to nanodomains of early endosomes that also contain ESCRT-0 and the ESCRT accessory factor HD-PTP, which collectively drive the sequestration of EGFR1 prior to its incorporation into ILVs.^7,10,38,39^ To examine the nanoscale relationship of Rab5a and endofin with EGF and EGFR1, we carried out a series of 3-color 10x ExM Airyscan imaging experiments. Alongside the fluorescently tagged EGF, we performed a four-way analysis between EGFR1, Rab5a, and endofin. Figure 4A,B illustrates overlays of EGFR1, EGF, and endofin in exemplar endosomes imaged at 15 and 30 min time points. A similar comparison between EGF, endofin, and Rab5a (Figure 4C,D) demonstrated that the two latter molecules remain at the endosome surface.

**Figure 4:**
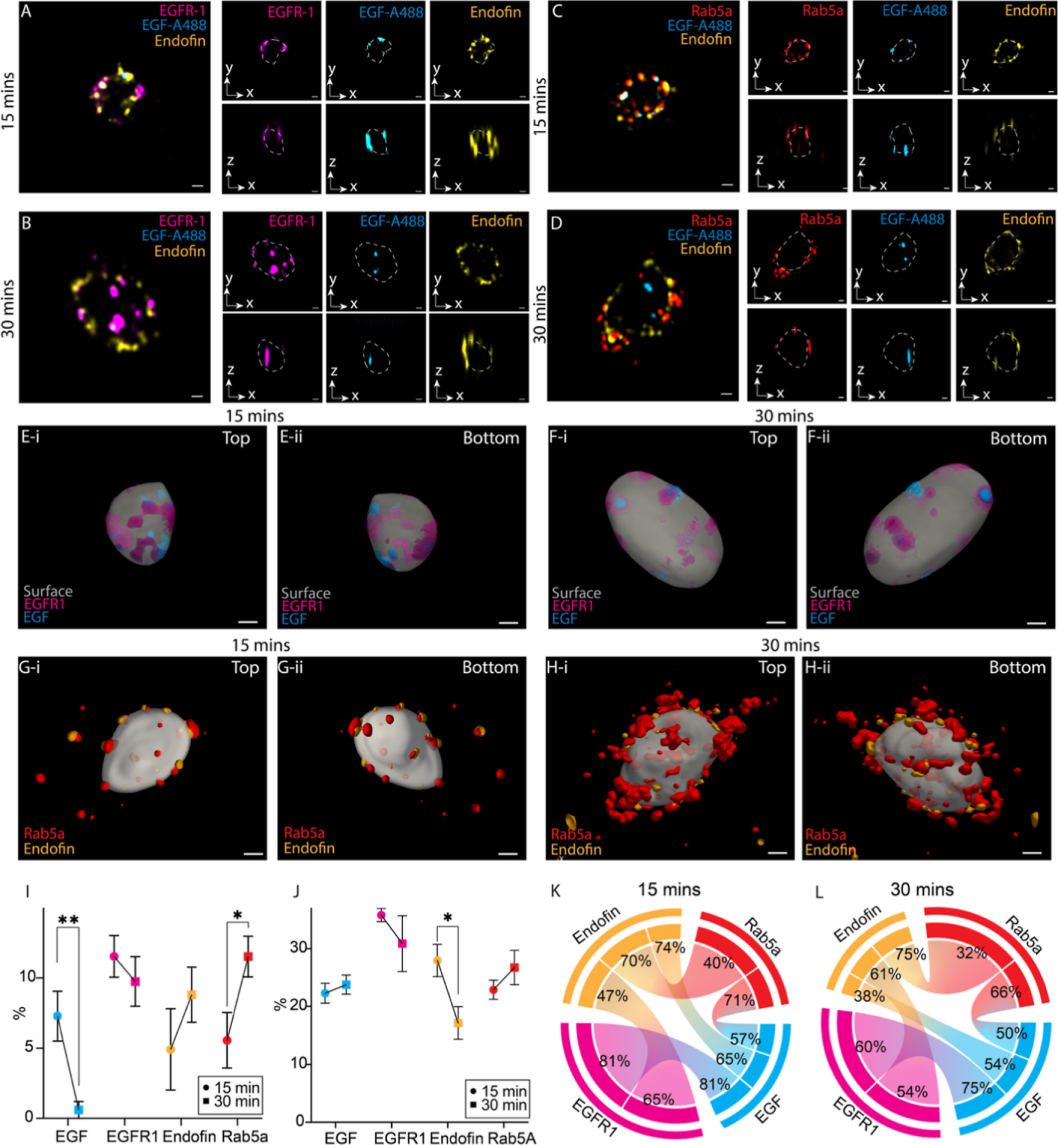
Visualization of the 3D organization of EGFR1 sorting proteins. Shown in the first comparison are three-color 10× ExM Airyscan image volumes overlaying EGF (cyan), EGFR1 (magenta), and endofin (yellow) labels in exemplar endosomes from (A) 15 min and (B) 30 min time points following the pulse-chase protocol. In the second comparison, equivalent image volumes overlaying EGF (cyan), Rab5a (red), and endofin (yellow) labels are shown at from (C) 15 min and (D) 30 min time points. In each example, individual channels of the volumes are shown in both in-focus *x*–*y* (upper row) and *x*–*z* (lower row) views. Top and bottom views of isosurfaces of volume-rendered examples of endosomes from (E-i,ii) 15 min and (F-i,ii) 30 min are shown. Translucent gray depicts the surface of the endosome traced based on endofin labeling, while EGF (cyan blue) and EGFR-1 nanodomains are projected onto the surface. Opaque and lighter-colored regions indicate the nanodomains in the reverse side of the endosome. Volume-traced endosomes from (G-i,ii) 15 min and (H-i,ii) 30 min time points isosurface-rendered to show the traced surface (translucent gray), endofin (yellow), and Rab5a (red), particularly its heterogeneous macro-clustering pattern at 30 min. (I) Line plots of the mean percentage of endosomal surface area occupied by projected EGF and EGFR1 nanodomains (as shown in panels (E) and (F)) as well as endofin and Rab5a nanodomains at 15 to 30 min time points. Error bars indicate SD **p < 0.0001, df = 7 data sets; *p < 0.001, df = 8 data sets. (J) Line plots of the mean percentage of pixels representing the cross-sectional area of the endosome occupied by each target of interest from 15 to 30 min. Error bars indicate SD *p < 0.05, df = 8. Chord diagrams summarizing the % of colocalization of a protein of interest with the reference protein at (K) 15 min and (L) 30 min time points. For example, the % of endofin markers overlapping with Rab5a at 15 min is 69.68%. The relative length of the arcs of each protein target of interest has been scaled in proportion to the 2D area occupied by the protein (summarized in panel (I)) at the corresponding time point. Scale bars: 100 nm.

To visualize the nanodomains occupied by these molecules, we developed a new method for tracing the endosomal volume that produced a model of its limiting membrane (see supplementary methods Section 3.6). This method used the punctate labeling densities that line the compartment’s limiting membrane to construct an iterative series of Delaunay triangulation transformations, which were then averaged to achieve a smoothed boundary of the endosome (Figures S14 and S15). In Figure 4E,F, the adjacent EGFR1 (magenta) and EGF (cyan blue) labeling (within ±40 nm of the boundary) were projected onto the traced endosomal surface (gray) to visualize the surface nanodomains they occupy (see Movie S1). A similar rendering of the endosomal surface shows the 3D labeling densities of both Rab5a (red) and endofin (yellow) are initially recruited to distinct nanoclusters on the endosome surface (at 15 min; Figure 4G). While the endofin puncta appeared to be relatively sparse at the endosomal surface at 30 min, larger densities (macro-clusters) of Rab5a were observable adjacent to the endosomal surface (Figure 4H and Movie S2).

Plots of the percentage of the endosomal surface area occupied by the projected EGF indicate a ∼90% reduction in density between 15 and 30 min (*p* < 0.01; df = 7 endosomes), particularly as the size and overall surface area of the endosome grow. A doubling of the percentage of endosomal surface area adjacent to the projected Rab5a is also seen, reflecting the enrichment of Rab5a into the macro-clusters observed above. The percentages of the endosomal cross-sectional area occupied by EGF, EGFR, Rab5a, and endofin at the two time points are plotted in Figure 4J. An ∼39% percent reduction in the density of endofin and an ∼17% increase in the density of Rab5a were observed. In the pairwise Manders colocalization analyses for 15 and 30 min summarized in Figure 4K,L respectively (see measured values in Table S1), a 21.2% reduction of the EGF colocalizing with EGFR is consistent with the reduction in the EGF/EGFR1 ratio of puncta in Figure 3. The reduction in endofin density observed at both the limiting membrane and the endosome cross-section between 15 and 30 min (Figure 4I,J) coincides with a 9% reduction of endofin labeling colocalizing with each of EGF and EGFR. In spite of the increase in Rab5a density and its accumulation near the limiting membrane between these two time windows, its colocalization with EGF and endofin is mostly unaltered.

### ExM as an Endosome Imaging Method

We report the adaptation of variants of widely used 4x and 10× ExM methods for imaging endosomes. The data presented here of 10× ExM combined with resolution-enhancing Airy-scanning microscopy achieve a level of spatial detail of the endosome that surpasses previous observations from localization modalities alone. In addition to the capacity to visualize both top and bottom surfaces of the endosomal vesicles, this has allowed us to localize, measure, and quantify the nanoclusters of EGF/EGFR1 complexes and signaling proteins contributing to the sorting process (Figures 3H–K and 4I–L). It is also the first 3D optical data that allow measurements of sequestered and/or enriched nanoclusters of these proteins within a small compartment. The much-improved 3D resolution with this approach has been pivotal for developing a new protocol for endosomal volume reconstruction and visualization of EGFR1 nanodomains at the limiting membrane (e.g., Figure 4E,F).

Measured nanoclusters provide an upper bound to the measurements of nanocluster size in the context of probe geometry. The Alexa-EGF signal is a compact ligand readout and therefore provides a comparatively direct estimate of ligand–receptor spatial distribution, whereas EGFR immunostaining can appear broadened by the size of primary/secondary antibody complexes and further biased by epitope accessibility (our antibody recognizes the luminal domain), particularly if receptors are densely clustered or being sorted into ILV-associated structures. While postexpansion labeling^40^ has emerged as a solution for minimizing spatial error, we have found that (i) the gains promised by this method (for example, the reduction in autofluorescence and improved antigenicity) are modest for structurally simple samples like RPE-1 cell monolayers, (ii) antigens may be lost if the anchoring and denaturation steps are not optimized appropriately, and (iii) it adds complexity to postlabeling washing steps in order to avoid nonspecific or diffusive background. More robust versions of ExM methods designed to reduce spatial error (e.g., Magnify or U-ExM).^19,25^ may offer more consistent matching between intrinsic and macro-scale EFs. However, we note that the 10-fold ExM data presented in Figure 2C featuring radial distortions around endosomes were acquired from an ExM recipe derived from a method with improved anchoring and gelation efficiency.^34^ The application of 3D error detection analysis is hence always advisable, in spite of the improved gel chemistries that are increasingly available to the cell biology community.

### Visualization of EGF Sorting Protein Interactions

Between 15 and 30 min, we observed a 10% reduction in the surface EGFR1 density, along with a 90% reduction in the density of EGF (Figure 4I). We also observed (i) an increase in EGF-EGFR1 puncta nearest neighbor distances (Figure 3I) and (ii) a modest (20%) decrease in the Manders colocalization measurements between EGF and EGFR (Figure 4K). The latter two observations may reflect features of the EGFR1 sorting process. The selective sequestration of ligand-bound EGFR1 receptors would enrich them within the interior,^41^ while it is also possible that some EGFR lacking Alexa-conjugated EGF may have acquired unlabeled EGF during the chase period.

Endofin, a key component of the EGFR1 sorting nanodomains at the limiting membrane, maintains a high colocalization (∼47–61%) with EGFR1 and remains at the membrane at 30 min (Figure 4I–L). In contrast, the colocalization of endofin with the fluorescently conjugated EGF reduces substantially (from 74% to 38%). The 38% reduction in the % of the endosome’s cross-sectional area occupied by endofin is explained in part by the increase in the endosomal size but may also reflect removal of endofin from the endosome as the sorting of EGFR1-EGF complexes to ILVs progresses. Rab5a continued to accumulate at the endosomal limiting membrane between the 15 and 30 min time points, forming macro-clusters at distinct regions of the endosomal surface, visually resembling the lipid-rich subdomains observed previously in HeLa cells with 2D dSTORM of Rab5c.^11^ While we did not measure a statistically significant change in the Rab5a puncta size, the observation of macro-clusters in ExM volume data and the doubling of its coverage over the approximated limited membrane support previous observations of continued Rab5 enrichment on the surface nanodomains.^42–44^ We anticipate the macro-clusters of Rab5a to be remobilized as the endosome continues to mature.

## New Tools for Quantitative ExM

### A Nondestructive and Nanoscale Reporter of Gel Expansion

Ten years on from the introduction of ExM, the need for improving the reliability and standardization of the post-ExM images, as well as their accurate interpretation, continues to be an intense area of focus. Existing methods of distortion detection or spatial analysis remain fundamentally limited by the spatial resolution of the reference (pre-ExM) images, limiting the method’s utility as a quantitative tool for imaging nanoscale structures. DNA origami^24^ has been used for this purpose previously in cell-free samples. The adaptation of DNA calibrants for cell imaging, however, would require anchoring chemistry compatible with both nucleic acids and proteins. A fundamentally protein-based calibrant such as this nanocage is a more straightforward and authentic reporter of protein-retaining gel expansion. Analysis of intrinsic cellular structures such as muscle z-discs, microtubules, and nuclear pore complexes^20,24,45^ is a popular method to quantify the intrinsic expansion isotropy or EF. A precharacterized calibrant such as the decahedral nanocage adopted here also provides a known ground truth of its dimensions, independent of cell type or physiology. The survivability of the GFP tag on each subunit during protein-retention ExM protocols^21,46^ makes them intrinsically detectable and measurable in post-ExM gels. As a well-characterized and self-assembling structure compatible with both mammalian and bacterial expression systems,^33,47^ this nanocage would be compatible with a wide range of ExM experiments. Once the cells were transfected, it required no additional modifications to the labeling, gelation, or expansion steps of the ExM protocol. Unlike recent methods of gel anisotropy detection that require physical alteration of the sample either through photobleaching^48^ or feature imprinting,^29^ a genetically encoded nanocage provides an entirely nondestructive approach to estimating the EF intrinsic to the structures and region of interest.

The 2D width of the nanocage was above the estimated in-plane resolution in the data following ∼10-fold expansion with methods such as ×10 ExM^27^ and TREx.^34^ This size match made this specific calibrant well suited for modest deviations in the intrinsic EF in the hydrogel. The nanocages were unresolvable when combined with 4× ExM and were measured to be similar in width to the Airyscan PSF (Figure S6), hence not suitable for calibrating the EF in 4× ExM samples unless it could be combined with a super-resolution image acquisition modality such as STORM.^49^ To verify why the hollow middle of the nanocages was not detectable in ExM gels, we performed a series of simulations (Figure S8), which revealed that this was due to the interaction of the top and bottom surfaces of the nanocage with the axially elongated PSF of the Airyscan PSF. We achieved the highest efficiencies in delivering the nanocages to the cell interior through cDNA transfection rather than premixing of purified nanocages with the gelation/monomer solution. A sparse, cytoplasmic distribution of the nanocages achieved this way was naturally the result of this expression approach. Due to the lack of compartment or cytoskeletal tether within the cell, some nanocages may be lost during the cell fixation or subsequent processing steps. The volumetric expansion of the cell during ExM (and the effective narrowing of the optical sectioning) also leads to a perception of sparsity in each Airyscan image plane. A greater density of nanocages could have provided a higher-resolution map of the gel isotropy. However, the natural sparsity observed in our samples was best suited for preventing aggregation and for avoiding cytotoxicity to the cell.

## Workflow Considerations for In Situ ExM of Endosomes

### Fixation and Nanocage Retention

Loss of diffusible proteins and complexes during formaldehyde fixation is well documented^50^ and can reduce the nanocage population retained throughout the experiment. For our key combination (Alexa488-EGF plus mouse *anti*-EGFR1), 2% paraformaldehyde (PFA; w/v; 10 min) was used to balance the antigenicity of endosomal proteins and expandability with protein-retention ExM recipes^51^ against better retention of diffusible nanocages. For non-ExM labeling within this probe combination, we also observed that 3% PFA and 3% PFA +0.05% GA performed similarly. Methanol fixation showed evidence of a diminished labeling density. While cross-linking enhancers like FixEL^52^ may be used in combination with PFA, they are not compatible with endogenous molecules such as the genetically expressed nanocage calibrant used in this study. Evaluating the combination of the target proteins/compartments of interest, material properties of the sample, and the hydrogel of choice is therefore advisable in the adaptation of ExM for visualizing subcellular structures such as endosomal protein complexes.

### Choice of Membrane Counterstains for Visualizing the Endosomal Limiting Membrane

We have demonstrated the potential use of the NHS version of BODIPY630 as a nondescript counterstain and of PI3P with GFP labeling as a targeted membrane reporter of endosomes (Figure 1I–K). High background fluorescence resulting from nonspecificity is a feature of BODIPY630, while the domain-forming biological function of PI3P results in a patchy or punctate morphology. The incorporation of membrane counterstains requires evaluation against the number of spectral channels available in multiplexed ExM imaging. The protein localization-based endosomal surface reconstruction approach (Supplementary Section 3.6) offers a useful analysis method in this context.

### 3D Distortion Mapping and 3D Reconstruction Tools

Distortion mapping through pre- and post-ExM imaging has been the primary method of benchmarking expansion isotropy since the inception of the ExM concept.^15^ Distortions resulting from expansion anisotropy are always three-dimensional. The 2D analyses available to date capture only the distortion components in the *x*–*y* planes. In our method, the integration of the components in the orthogonal planes was critical for our discovery of the radial nature of misregistrations local to some endosomes. For all users adopting ExM, irrespective of single-plane or volume imaging, this approach offers a more robust benchmarking tool than the 2D methods.

One of the remaining limitations of ExM is the limited repertoire of reliable membrane labels specific to small intracellular compartments that can survive gel expansion. In the endosomal volume reconstruction, endofin was used as an antigen marker of the limiting membrane. Similar to many membrane marker antigens, endofin’s punctate morphology only lends a discontinuous impression of the membrane. Our volume reconstruction approach of iteratively detecting and fitting puncta and projecting them onto a triangulated surface has helped us transform a relatively sparse labeling pattern into a continuous volumetric representation of the vesicle. This method offers a scalable pipeline that can be translated and applied to ExM volumes of other numerous types of organelles or cells. In connection to endosomes, however, this method carries two principal limitations. As an inherently smoothing-based approach, this algorithm is unsuitable for reconstructing finer topologies such as membrane tubules or invaginations unless they are discernible with a specific marker. Without specific markers, we were also unable to reconstruct intraluminal vesicles (ILVs) that carry out the sequestration of the EGF/EGFR1 complexes.

## Conclusions

The combination of 10× ExM with a new 3D visualization toolkit allowed us to observe the time-dependent evolution of EGF/EGFR1 interactions with early endosomal regulators and to resolve distinct limiting-membrane architectures that had not previously been accessible with optical microscopies. The adoption of genetically encoded icosahedral protein nanocages as a calibrant for 10× ExM allowed us to detect, measure, and correct under-expansion in cytoplasmic regions that could otherwise systematically overestimate geometry and distance measurements for small compartments such as endosomes. Collectively, these advances established ExM as a more reliable platform for interrogating subcellular organelles, unlocking routine, quantitative nanoscale mapping of protein complexes and surface domains associated with some of the smallest membrane structures in cells.

## Methods/Experimental Section

### Cell Culture

Human retinal pigment epithelial RPE-1 cells were cultured in DMEM/F-12 Ham liquid with l-glutamine and sodium bicarbonate (Gibco D8062, Merck, UK) and 10% fetal bovine serum (FBS; v/v; Cat. no. 17563595 Fisher Scientific Ltd., MA), supplemented with 1% penicillin–streptomycin (v/v; containing 5000 units/mL of penicillin and 5000 μg/mL of streptomycin; Thermo Fisher, MA). For transfections in RPE-1 cells, penicillin–streptomycin was omitted from the cell culture medium for >1 passage. HeLa cells were cultured in DMEM supplemented with 10% fetal bovine serum and supplemented with 1% penicillin–streptomycin. Cells were incubated at 37 °C and 5% CO_2_ and seeded once they reached ∼70–80% confluency, typically 2 days after passage. After removing the excess, cells were washed with PBS buffer and detached from flasks with 0.05% trypsin–EDTA (Cat. no. 25300054, Fisher Scientific), and trypsin was neutralized with complete fresh medium.

Cells were seeded onto No. 1.5 glass coverslips (Epredia ×1000 cover glasses 22 × 22 mm # 1.5 (0.16–0.19 mm), Cat. no. 15805214, Fisher Scientific) in 6-well plates at a density of 50,000 cells/ml (2 mL of the passaged mixture). Seeded cells were incubated in cell culture medium at 37 °C and 5% CO_2_ for ∼2 days, until they reached ∼70–80% confluency. Intrinsic calibration of ×10 ExM was performed in RPE-1 cells seeded at a density of 20–25,000 cells/ml (1 mL of the passaged mixture per well) and incubated for ∼2–3 days before transfection at ∼20–40% confluency. Cell culture medium was replaced with fresh complete medium (2 mL per well) > 4 h before transfection or pulse-chase experiments.

### EGF Pulse Chase

RPE-1 cells were cultured on coverslips to ∼70% confluency for ∼2 days as discussed above. Cells were serum-starved for ∼4 h in serum-free DMEM at 37 °C 8% CO_2_. Cells were then incubated with 50 ng/μL EGF conjugated by a streptavidin linker to Alexa-488 (final [EGF] = 5 ng/μL) for 5 min at 37 °C 5% CO_2_, and coverslips were immediately transferred to serum-free medium containing 50 ng/μL unlabeled EGF. Cells were incubated for 15 or 30 min before fixation and immunostaining.

### Expression of Decahedral Nanocages in Cultured HeLa and RPE-1 Cells

The I3-01 sequence encoding a single monomer subunit of the self-assembling 60mer nanocage (Table 1) developed by David Baker’s laboratory^33^ was cloned into a mammalian expression vector transfected for expression in RPE-1 cells. Cells were cultured and seeded onto coverslips and maintained until they reached ∼20–40% confluency. They were transfected with plasmid DNA using lipofectamine per the manufacturer’s instructions and optimized for cell viability and nanocage expression levels.

**Table 1:** Details for cDNAs Used to Express Nanocages in Mammalian Cells^a^

cDNA—gene name	protein encoded	molecular weight (kDa)	vector used for cloning	sequence
I3-01_pEGFP_NTERM	monomer with 1 nt eGFP for 60mer decahedral nanocage	55.72	pcDNA3.1(+)-*N*-eGFP	GGATCCGGAGGAAGCGGAGGATCTGGCGGCAGCGGCGGCAGCGGCAAGAT​TGAGGAACTGTTCAAGAAGCATAAGATCGTGGCCGTGCTGCGGGCCAATAG​CGTGGAAGAGGCCAAGAAAAAGGCCCTGGCTGTTTTCCTGGGCGGAGTGCA​CCTGATCGAGATCACCTTCACCGTGCCTGATGCCGACACCGTCATCAAGGA​ACTGAGCTTCCTGAAGGAAATGGGCGCCATCATCGGCGCCGGCACCGTGA​CAAGCGTGGAACAGTGC
				AGAAAGGCCGTCGAGAGCGGCGCTGAGTTCATCGTGTCCCCTCACCTGGAC​GAGGAAATCAGCCAGTTCTGCAAGGAGAAGGGTGTGTTCTACATGCCTGGCG​TCATGACCCCTACAGAGCTCGTGAAGGCCATGAAGCTGGGCCACACCATCC​TGAAACTGTTTCCCGGCGAGGTGGTGGGCCCCCAGTTTGTGAAAGCCATGAA​AGGCCCTTTCCCCAACGTGAAGTTCGTGCCAACCGGCGGCGTGAACCTGGA​CAACGTGTGCGAG
				TGGTTCAAGGCTGGCGTGCTGGCCGTGGGCGTTGGCTCCGCCCTGGTGAAGG​GCACACCTGTGGAAGTGGCCGAGAAGGCTAAAGCCTTTGTGGAAAAGATCA​GAGGCTGTACAGAGGGCTCTGGATCTGGCAGCGGAAGCGGCTCCTGAGAAT​TC

a The name, molecular weight, and sequence are provided for the mammalian expression vector for nanocage expression.

Cells were transfected with 0.5 μL of plasmid DNA and 0.5–1.5 μL of lipofectamine 2000 (Cat. 11668019 Thermo Fisher) per 10 cm^2^ well containing 2 mL of cell culture medium. For each well, 0.5 μg plasmid DNA was gently mixed with Opti-MEM medium (cat. 31985062, Thermo Fisher) to a final volume of 125 μL. A 0.5 μL or 1.5 μL portion of lipofectamine 2000 reagent was mixed gently with Opti-MEM medium to a final volume of 125 μL. DNA and lipofectamine dilutions were incubated for >5 min at room temperature before mixing them together by gentle pipetting, and the resulting transfection mixture was incubated for >15 min at room temperature. A 250 μL portion of the transfection mixture was added dropwise to plated cells in 2 mL of cell culture medium in a single well of a 6-well plate as described above. After 20–24 h of transfection, cells were fixed and either stained with DAPI (diluted to 1 μg/mL in 1× PBS) for immunofluorescence imaging or expanded in ExM.

The DNA, PLUS reagent, and lipofectamine LTX or lipofectamine 2000 concentrations were adjusted in parallel, depending on cell confluency. All transfection conditions gave similar results that varied between the cells. Higher concentrations of transfection reagents improved expression but resulted in aggregation of nanocages in some cells, and lower concentrations resulted in even expression in some cells, but expression was limited in some cells. Cells were chosen for analysis based on the even expression of nanocages in the cell.

### Purification and Characterization of Nanocages from Mammalian Cell Lysate

HeLa cells expressing nanocages were washed with ice-cold PBS (1×), and 1 mL of ice-cold lysis buffer (1× PBS +10 mM EDTA +1× protease inhibitor cocktail (Cat. S8830, Merck Life Sciences, Darmstadt, Germany) + 1% Triton-X (Merck)) was added to cells plated on each coverslip. Lysed cells were scraped using a pipet tip and moved into centrifuge tubes. Lysates were kept on ice for 30–45 min with intermittent mixing by inverting. Lysates were spun down at 13,300 rpm (17,000*g*) for 20 min at 4 °C. Supernatant was removed and concentrated through a 1MDa spin concentrator (Vivaspin 500, Merck) and stored at 4 °C until imaging with electron microscopy.

### Immunostaining

Following transfection or pulse-chase experiments, coverslips were transferred to 2% PFA (v/v; 4% PFA solution in PBS was mixed 1:1 with 1% PBS to make up the final concentration of 2%; Fisher Scientific Ltd.) for 10 min at room temperature. Cells were washed 3× in 1% PBS to wash off excess PFA before quenching with ∼3–4 drops of 1 M glycine in *Tris* buffer or 0.5% BSA (w/v, Gibco, Thermo Fisher Scientific) in a cell storage solution consisting of 1% PBS (tablets dissolved in dH_2_O; Fisher Scientific).

Following fixation, cells were stored in PBS containing 0.5% bovine serum albumin (w/v) or PBS with ∼3–4 drops of 1 M glycine in *Tris* buffer at 4 °C until immunostaining steps. Before immunostaining, cells were permeabilized in PBS (137 mM NaCl, 2.7 mM KCl, 10 mM Na_2_KPO_4_, 2 mM KH_2_PO_4_, pH 7.3) + 0.1% (v/v) Triton X-100 and subsequently blocked in PBS +10% normal goat serum (NGS; v/v) + 0.05% Triton X-100.

Immunostaining was carried out in an antibody incubation buffer (0.05% Triton and 2% NGS in PBS), and coverslips were either attached to microscope slides with cut-out windows, creating a small well where 200 μL of antibody solution (Table 2) was added, or inverted on top of 100 μL of antibody solution on a parafilm-covered glass slide. Primary antibodies were added to coverslips and incubated overnight at 4 °C. The samples were then washed with fresh PBS 3 times in 10–20 min steps prior to secondary antibody application for 1.5 h at room temperature. The samples were then washed in fresh PBS 3 times in 10–20 min steps.

**Table 2:** Antibodies and Fluorescent Probes Used for Sample Labeling

primary antibodies	catalogue number	source	dilution
anti-EEA1 (mouse)	610457	BD Biosciences, CA	1:200
anti-Rab5A (mouse)	66339-1-Ig	Proteintech, IL	1:200
anti-Rab5A (rabbit)	11947-1-AP	Proteintech, IL	1:200
anti-endofin (rabbit)	13118-2-AP	Proteintech, IL	1:200
anti-EGFR1 (mouse)	Mab 108	purified from hybridoma clone HB-9764 ^53^	1:200
anti-HRS (rabbit)	Mab 15087	Cell Signaling Technology	1:100
anti-GFP Alexa568 nanobody	gb2AF568	Chromotek	1:200

secondary antibodies	catalogue number	source	dilution
Alexa Fluor 488 goat antirabbit	A11008	Thermofisher Scientific	1:200
Alexa Fluor 488 goat antimouse	A11001	Thermofisher Scientific	1:200
Alexa Fluor 594 goat antirabbit	A11012	Thermofisher Scientific	1:200
Alexa Fluor 594 goat antimouse	A11005	Thermofisher Scientific	1:200
Atto 647 N goat antirabbit	40839-1 ML-F	Merck Life Sciences	1:200
Atto 647 N goat antimouse	50185-1 ML-F	Merck Life Sciences	1:200

### BODIPY Counter-Staining of Endosomal Membranes

After permeabilizing the cells, 200 μL of BODIPY 630 NHS ester (Cat. D2191, Thermofisher) diluted 1:1000 in a staining solution (100 mM NaHCO_3_ + 1 M NaCl, pH 6, made up to 100 mL with dH_2_O) was added per well for 1 h 30 min at room temperature. Samples were washed 3 times with PBS in 20 min steps prior to the blocking step.

### PI3P-Targeted Counter-Staining of Endosomal Membranes

After 4 h of serum starvation, RPE-1 cells were fixed with 2% PFA for 15 min. Cells were quenched by washing 3 times with 50 mM NH_4_Cl in 1× PBS and permeabilized for 5 min with 20 μM digitonin in buffer A (20 mM PIPES pH 6.8, 137 mM NaCl, 2.7 mM KCl). Cells were washed 3× with buffer A and then blocked and labeled at the same time in buffer A containing 50 mM NH_4_Cl and 5% NGS with 1 μg/mL of a custom-made PI3P-binding tag (2xFYVE domain protein with a GST and GFP domain) for 45–60 min. Cells were washed 3× in buffer A and labeled with primary antibodies (diluted in buffer A + 5% NGS) for 45–60 min. After 3× washes with buffer A, cells were labeled with secondary antibodies (diluted in buffer A + 5% NGS) for 45–60 min. Cells were then washed 3× in buffer A and fixed again with 2% PFA for 5 min. Fixation was then quenched by washing 3× with 50 mM NH_4_Cl in 1× PBS, and cells were then anchored for subsequent expansion experiments or imaged directly. All fixation and labeling experiments were performed at room temperature.

The anti-PI3P label was generated as a recombinant protein expressed and purified from E. coli using standard techniques. GST-GFP-2xFYVE and GST-2xFYVE plasmids were generated by HiFi assembly (NEB, E5520), using pEGFP-2xFYVE as a template (a gift from Harald Stenmark, Addgene plasmid no. 140047; RRID/Addgene_140047).

### Expansion Microscopy

#### Anchoring

Fluorescently labeled samples were prepared for linking into the gel by incubating them at 4 °C overnight in 0.1 mg/mL Acryloyl-X (w/v; catalogue number A20770, Thermo Fisher Scientific) diluted in 1× PBS.

#### X10 Gelation

Cells in each well were washed with PBS 3 times in 20 min steps. A gel monomer solution consisted of a 4:1 molar ratio of dimethylacrylamide and molecular-grade sodium acrylate, dissolved in deionized H_2_O saturated in N_2_ over 1 h on ice. Potassium persulfate (KPS; Sigma-Aldrich Ltd., MO) was added at 0.4% molar relative to monomer concentration from a 0.036 g/mL stock, made fresh for each experiment, and the solution was bubbled for another 15 min on ice. A 500 μL portion of the gel monomer solution was mixed rapidly with 2 μL of TEMED and quickly added to the cells. Gelation was allowed in a sealed acrylic chamber comprising two coverslip walls with the coverslip with cells inverted on top of a gel solution in between these cell walls. Gel polymerization was completed by >6 h before the gels were extracted carefully from the chambers prior to the digestion step.

#### Digestion and Expansion

Gels were subjected to digestion in 0.2 mg/mL proteinase K (ProK, New England Biolabs, MA) dissolved in a “digestion buffer” (50 mM Tris pH 8.0, ThermoFisher; 1 mM ethylenediaminetetraacetic acid (EDTA; Sigma); 0.5% Triton X-100; 0.8 M guanidine HCl (Sigma), in deionized H_2_O) for 10–13 h at RT on an orbital shaker. The gels were washed 3× with PBS for 5–10 min at room temperature on an orbital shaker. Gels were either transferred directly to Petri dishes for expansion or stained with a 1 μg/mL solution of DAPI in PBS for 15–20 min, followed by 3 × 10 min washes in PBS at room temperature on an orbital shaker. Gels were transferred to a large glass Petri dish where they were expanded by gentle washing in fresh deionized H_2_O for 30–60 min, repeated ∼4 times, until there was no further change in the gel size.

The TREx variation of the ExM protocol is detailed in Supplementary Methods section 3.5.

#### Variation in the 4× ExM (proExM) Expansion Protocol

The gelation solution was prepared as described previously.^21^ Cell samples were incubated with the monomer solution (w/v, Sigma-Aldrich) of 8.6% sodium acrylate, 2.5% acrylamide, 0.15% *N,N′*-methylenebis­(acrylamide), 11.7% NaCl, PBS, 0.1% ammonium persulfate, and 0.1% *N,N,N′,N′*-tetramethylethylenediamine first for 30 min at 4 °C and then for 2 h at 37 °C.

#### Microplate-Based ExM Sample Preparation and Pre-/Post-ExM Imaging

The 3D-printed square-well microplates and the laser-cut spacers (described previously)^26^ were used for experiments performed for pre- and post-ExM analysis. Cells were cultured at the center of the 20 × 20 mm square wells. Following fixation and immunolabeling, the spacers were sealed onto the well, leaving a 5 × 5 mm central gap within which the gelation and digestion steps were performed. The spacer was removed prior to hydration of the square well, ensuring that the gel expands without any rotation. In 10-fold ExM experiments, the gel was trimmed down to retain an ∼2 mm × 2 mm corner. The *x*, *y*, and *z* stage coordinates of the cells of interest during pre-ExM imaging were used to calculate and relocate the structure after expansion (see protocol provided by Seehra et al.;^26^ Methods 3.1).

#### Airyscan Microscopy

For imaging ExM samples, the gels were placed within acrylic chambers, which were custom-made to fit the stages of the microscopes. The chamber itself was typically square (adapted in size and shape to fit 4× or 10× ExM gels) and consisted of a base made of a glass No. 1.5 coverslip (Menzel Gläser, Germany), custom-coated with 0.1% (v/v) poly-l-lysine (Sigma) in order to retain the gels flush on the coverslip.

All images were obtained on a Zeiss LSM980 AiryScan2 (Carl Zeiss, Jena, Germany) with a Zeiss 40 × 1.3 NA oil-immersion Plan Apochromat objective. Imaging was performed in Airyscan mode with the gain and laser power adjusted for each sample to accommodate the fluorescence reduction occurring due to the spatial separation of fluorophores during expansion. Fluorophores were excited using Argon 405 and 488 and DPSS 561 and 642 nm laser lines. Image data was acquired using the Zen Blue interface that allowed us to select the emission bands to minimize spectral cross-talk between dyes. In Airyscan mode, emission was recorded onto the GaAsP detector, and the data were subjected to postacquisition pixel reassignment analysis (with the essential, built-in Wiener deconvolution)^31^ with Zen Blue as described previously.^20^

#### Negative Stain Transmission Electron Microscopy of Nanocages

Palladium-coated copper grids coated with carbon film were preprepared by the electron microscopy facility team at the University of Sheffield. Carbon film-coated grids were glow discharged for 20–25 s and incubated with 3–5 μL of various concentrations of protein sample for 5–30 s. Grids were blotted and washed 2× by dipping in dH_2_O and blotting. Grids were stained with uranyl formate by a brief wash followed by 20 s incubation and then dried before imaging.

Negatively stained samples were imaged with TEM and were examined using a Philips CM100 transmission electron microscope or the Tecnai 12 transmission electron microscope. Magnification was set to 23,000×. Images were captured using a CCDC camera. The focus was defocused slightly according to the sample to improve image contrast.

### Image Analysis

#### Pre- and Post-ExM Imaging and Distortion Analysis

The pre- and post-ExM image volumes were registered to each other using an iterative block-matching analysis pipeline implemented via the plugin Fujiyama via ImageJ (see Supporting Information Section 3.2 for details). A series of aligned 2D slices from *XY* and *XZ* planes throughout the image volumes were then subjected to a Farneback optical flow analysis, described in the Supporting Information, Section 3.3, which produced distortion shift vectors for each 2D slice considered. Pairing up the *XY* and *XZ* shift coordinates for a given reference point allowed us to recalculate the 3D distortion vectors (see visualization shown in Figure 2C-iii,iv, produced with open-source Mayavi). The analysis and visualization code is provided in the data supplement.

#### Analysis of Nanocages in Negative Stain TEM Image Data

Nanocage diameter was determined from electron micrographs. An elliptical boundary was manually defined for each nanocage (as illustrated in Figure 2F inset). The minor and major axes of each ellipse were determined using the “Measure” function in ImageJ. Nanocage width was determined from the average of the minor and major axis measurements.

#### Detection and Analysis of Nanocages in Airyscan Image Data

2D Airyscan image data (unexpanded and post-ExM) were analyzed with Python microscopy environment (PyME; downloaded from python-microscopy.org on 11/11/2024). See Supplementary Methods Section 3.4 for details.

#### Measurement of Endosomal Widths in 10× ExM Image Data

Image volumes were imported into ImageJ, and the pixel scale was adjusted to account for either the estimated macroscopic or intrinsic EF. In z-stacks, images where endosomes were in focus were selected, and the ellipse function was used to manually fit the torus-shaped endosomes. The Measure function was then used to read out the major and minor axes of the ellipse, and the average of these two values was recorded as the width.

#### Chord Plots for Displaying Marker Density and Colocalization

A custom R script (included in the data supplement) was developed to summarize the colocalization measurements in a chord diagram (Figure 4K,L). In each plot, the length of the circumference was subdivided into arcs assigned to a given protein in proportion to the average proportion of the pixels occupied by the marker at a given time point (as displayed in Figure 4E). The chords were plotted such that the percentage noted at the near end of each chord reported the percentage of that marker colocalizing with the marker at the far end.

#### Resolution Estimation with PSF Measurements

To obtain PSF estimates, 3D stacks were taken of 100 nm multispectral polystyrene Tetraspeck Fluorescent microspheres (T7279, Thermo Fisher, UK) suspended in acrylamide. The lateral resolution of the LSM 980 Airyscan 2 system was measured to be ∼170 nm.

#### Analysis of Endosomal Features ExM Image Data

All puncta localization, nearest neighbor distance, and size analyses were performed in Huygens software (SVI, Hilversum). Manders colocalization percentages^54^ were calculated using the coloc2 plugin in FIJI (ImageJ v1.54f) with the BioFormats plugins. Binary masks for the colocalization analysis were constructed by automatic thresholding using the Moments thresholding option in FIJI. All statistical tests on measurements reported in this paper were nonparametric and were performed in GraphPad Prism. Open-source software ParaView v6.0.0 was used for 3D isosurface visualization of data.

The volume of endosomes was traced using a surface protein localization approach applied to 3D 10× Airyscan image stacks of endofin (detailed in Supporting Information, Section 3.6). This leveraged the observation that endofin appeared to closely outline the endosomes on the cytoplasmic side.

## Supplementary Material





## Data Availability

This manuscript was previously made available publicly as a preprint (see ref 56).

## Notes

Supplementary figures, captions for the supplementary movies, and supplementary methods The analysis code and exemplar supplementary data are provided at an online repository accessible via online repository (see ref 55) (PDF)
Surface-rendered view of endosomal localization of EGF and EGFR1 and surface-rendered view of endosomal localization of Rab5a and endofin (ZIP)
